# Abbreviated version of the Bladder Health Scales for women’s research

**DOI:** 10.1016/j.ajog.2025.10.015

**Published:** 2025-10-17

**Authors:** Todd Rockwood, Kyle Rudser, Leslie Rickey, Alayne D. Markland, Peter Scal, Jerry L. Lowder, Emily S. Lukacz

**Affiliations:** Division of Health Policy & Management, University of Minnesota School of Public Health, Minneapolis, MN; Division of Biostatistics & Health Data Science, University of Minnesota School of Public Health, Minneapolis, MN; Departments of Urology & Obstetrics/Gynecology, Yale University School of Medicine, New Haven, CT; Department of Medicine, Division of Gerontology, Geriatrics and Palliative Care, University of Alabama at Birmingham, Birmingham, AB; Division of Health Policy & Management, University of Minnesota School of Public Health, Minneapolis, MN; Department of Obstetrics and Gynecology, Washington University in St Louis School of Medicine, St Louis, MO; University of California San Diego, Department of Obstetrics, Gynecology & Reproductive, Sciences, La Jolla, CA

**Keywords:** bladder, function, health, index, lower urinary tract symptoms, measurement, refinement, scales

## Abstract

**OBJECTIVES:**

The Bladder Health Scales (BHS) 10 and Bladder Function Index (BFI) were developed and validated to assess the distribution of bladder health from poor to optimal among community-dwelling women.^1–3^ The BHS-10 evaluates the impact of the bladder on various aspects of health and wellbeing including an adaptive behaviors adjustment (ABA) and the BFI measures 6 elements of bladder function. We aimed to identify a shorter version of the BHS to decrease participant burden yet provide meaningful assessments of bladder health for use in population-based longitudinal studies.

**STUDY DESIGN:**

A prospective observational study of a random sample of community-dwelling females in 57 counties surrounding 9 US research sites was conducted.^4^ Participants completed the BHS-10 with ABA and BFI at baseline and again 1 year later. Analytically, 4 criteria were used to identify candidate scales for the abbreviated instrument: (1) retention of psychometric properties relative to the original instrument validation using Cronbach’s Alpha and Factor Analysis^1^, (2) responsiveness to change relative to changes in the Lower Urinary Research Network-Symptom Index 10 bother question^5^ and total BFI scores, (3) discriminative ability as measured by receiver operator curve area under the curve for a single item summary of “better” vs “worse or same” from a year ago, and (4) content validity assessed by experts confirming relevant scales for longitudinal assessments.

**RESULTS:**

Of the 3375 with baseline data, 2310 (68%) completed 1-year follow-up. Psychometric screening demonstrated retention of internal relationships; however, replication of factor analyses identified 3 scales (Social-occupation, Travel, and Emotional) with inconsistent factor loading relative to the original instrument validation^1^ (Table 1). Table 1 demonstrates the change scores for the remaining 7 scales compared against the change scores in the Lower Urinary Research Network-Symptom Index 10 bother (RSq=0.09) and change in total BFI (RSq=0.25) where the Global and Holding retained statistically significant (*P*<.05) betas. The receiver operator curve area under the curve for each scale calculated against overall change in perceived bladder health from baseline (better vs worse/same) were all between 0.54 and 0.59 (Table 1). Linear regression results (Table 1) demonstrate that the difference in variance explained in the outcomes did not vary dramatically between the 2, 3, and 7 scale instruments. The final step in scale retention selection rested on evaluation of the content of the scales by clinical experts who identified the Global, Holding, and Efficacy scales as being most relevant for community surveillance studies.

**CONCLUSIONS:**

We present a systematically developed, valid, abbreviated version of the BHS (BHS-3) to be used along with the ABA and BFI for community-based epidemiological cross-sectional and longitudinal research in women. The BHS-3 is 68% shorter than the BHS-10 (from 38 to 12 individual questions) which should decrease the time for completion to 4 to 6 minutes. While researchers may prefer the BHS-10 to measure bladder health related to a wide range of day-to-day activities and emotional factors, others may prefer the BHS-3 with ABA as a meaningful surveillance tool to evaluate 3 core measures of bladder health along with the bladder function. These instruments are free for use without special permission.

## Supplementary Material

1

## Figures and Tables

**TABLE 1:** Findings from models run associated with shortening the BHS

Part 1a: psychometric evaluation							Part 1b: reduction evaluation using change (Δ) scores				
Scales	Cronbach’s alpha			Factor analysis Eigen values			Δ LURN-SI bother		Δ total BFI score		Δ overall BH^a^
	VIEW	RISE Baseline	RISE 1 y follow-up	VIEW	RISE Baseline	RISE 1 y follow-up	Model RSq	Scale Δ Beta	Model RSq	Scale Δ Beta	Scale Δ ROC
#1 Global	0.91	0.91	0.89	4.2	4.1	3.9	0.09	0.09	0.25	0.24	0.59
#2 Holding	0.80	0.81	0.76	2.9	3.1	3.0		0.11		0.13	0.59
#3 Efficacy	0.76	0.77	0.76	1.4	1.2	1.2					0.57
#4 Social occupation	0.94	0.95	0.94	7.7	Fail	Fail					
#5 Physical activity	0.93	0.88	0.89	1.8	1.8	1.9					0.55
#6 Intimacy	0.88	0.93	0.93	1.3	1.2^b^	1.4				−0.11	0.54
#7 Travel	0.90	0.84	0.83	1.2	Fail	Fail					
#8 Emotion	0.91	0.92	0.90	6.2	Fail	Fail					
#9 Perception	0.93	0.93	0.94	1.2	5.7	1.7		0.08			0.57
#10 Freedom	0.74	0.75	0.70	1.1	0.9^b,c,d^	1.0^b^					0.55
Part 1c: comparison of retained 2, 3, 7 BHS											
						Δ LURN-SI bother RSq					Δ BFI RSq
2 scale (#1 & #2)						0.08					0.12
3 scale (#1, #2, & #3)						0.09					0.12
7 scale (#1-3, 5, 6, 9, 10)						0.09					0.13

Only significant results are shown. Betas for Intimacy and Perception were not significant across both Δ LURN-SI bother and Δ Total BFI, thus not included in subsequent analysis.

*BFI*, Bladder Function Index; *BH*, Bladder Health; *BHS*, Bladder Health Scales; *LURN-SI*, Lower Urinary Research Network-Symptom Index; *ROC*, receiver operator curve; *RISE*, RISE for Health Study; *VIEW*, The validation of bladder health instrument for evaluation in women study.

Fail reasons: Eigen < 1.0.

a BH item: Compared to a year ago, is your bladder function… Much better now, Somewhat better now, About the same, Somewhat worse now, Much worse now.

b Parallel analysis indicates scale is suspect.

c Factor loading for items do not meet 0.60/0.40 or 0.20 guidelines for inclusion.

d Items loading on wrong factor.

## Research centers and investigators:

Loyola University Chicago, Maywood, IL (U01DK106898): Multi-Principal Investigators: Linda Brubaker, MD; Colleen Fitzgerald, MD, MS. Investigators: Marian Acevedo-Alvarez, MD; Cecilia T. Hardacker, MSN, RN, CNL; Jeni Hebert-Beirne, PhD, MPH. University of Chicago, Chicago, IL (U01DK126045): Multi-Principal Investigators: James W. Griffith, PhD; Kimberly Sue Kenton, MD. Co-investigator: Margaret Mueller, MD. Project Manager: Melissa Marquez. Northwestern University, Chicago IL (U01DK126045): Multi-Principal Investigator: Melissa Simon, MD, MPH. Co-Investigators: Oluwateniola Brown, MD; Julia Geynisman-Tan, MD. University of Alabama at Birmingham, Birmingham, AL (U01DK106858): Multi-Principal Investigators: Alayne D. Markland, DO, MSc; Camille P. Vaughan, MD, MS. Investigators: Tamera Coyne-Beasley, MD, MPH, FAAP, FSAHM; Kathryn L. Burgio, PhD; Cora E. Lewis, MD, MSPH; Gerald McGwin, Jr., MS, PhD; Beverly Rosa Williams, PhD. University of California San Diego, La Jolla, CA (U01DK106827): Principal Investigator: Emily S. Lukacz, MD; D. Yvette LaCoursiere, MD, MPH. Investigators: Sheila Gahagan, MD, MPH; Jesse Nodora, DrPH. University of Michigan, Ann Arbor, MI (U01DK106893): Principal Investigator: Lisa Kane Low, PhD, CNM, FACNM, FAAN. Investigators: Janis M. Miller, PhD, APRN, FAAN; Abby Smith, PhD. University of Minnesota (Scientific and Data Coordinating Center), Minneapolis, MN (U24DK106786): Multi-Principal Investigators: Gerald McGwin, Jr., MS, PhD; Kyle D. Rudser, PhD. Investigators: Sonya S. Brady, PhD; Cynthia S. Fok, MD, MPH; Bernard L. Harlow, PhD; Peter Scal, PhD; Todd Rockwood, PhD. University of Pennsylvania, Philadelphia, PA U01DK106892): Multi-Principal Investigators: Diane K. Newman, DNP; Ariana L. Smith, MD. Investigators: Amanda Berry, MSN, CRNP; Andrea Bilger, MPH; Terri H. Lipman, PhD; Heather Klusaritz, PhD, MSW; Ann E. Stapleton, MD; Jean F. Wyman, PhD; Kristine Talley, PhD, RN, GNP-BC. Washington University in St. Louis, Saint Louis, MO (U01DK106853): Principal Investigator: Siobhan Sutcliffe, PhD, ScM, MHS. Investigators: Aimee S. James, PhD, MPH; Jerry L. Lowder, MD, MSc; Melanie R. Meister, MD, MSCI. Yale University, New Haven, CT (U01DK106908): Principal Investigator: Leslie M. Rickey, MD, MPH. Investigators: Deepa R. Camenga, MD, MHS; Shayna D. Cunningham, PhD. Steering Committee Chair: Linda Brubaker, MD. UCSD, San Diego. (January 2021). NIH Program Office: National Institute of Diabetes and Digestive and Kidney Diseases, Division of Kidney, Urologic, and Hematologic Diseases, Bethesda, MD. NIH Project Scientist: Jenna Norton, PhD, MPH.
