# Supplementary material for: Abbreviated version of the Bladder Health Scales for women’s research
Source: Am J Obstet Gynecol. Author manuscript; Available in PMC 2026 Jul 6. (PMC13335158; doi:10.1016/j.ajog.2025.10.015)
Supplement: 1 [file NIHMS2184815-supplement-1.pdf]

## APPENDIX

**Supplemental material 1 — Survey instrument: BHS-3 with ABA & BFI**
Abbreviated Bladder Health Scales (BHS-3)

1. When was the last time you thought about your bladder?
   - ☐ (6) Hardly ever, I can't remember the last time
   - ☐ (5) At least a month or longer
   - ☐ (4) Within the past week
   - ☐ (3) At least once today
   - ☐ (2) Within the past few hours
   - ☐ (1) In the past hour
2. Which of the following best captures how you feel about your bladder?
   - ☐ (6) It should be in the Bladder Hall of Fame/It's great
   - ☐ (5) I have a good one
   - ☐ (4) It works well enough
   - ☐ (3) It's not great
   - ☐ (2) I wish I could return it
   - ☐ (1) I got a lemon/I want a new one
3. My bladder is…
   - ☐ (5) No bother at all
   - ☐ (4) A little bothersome
   - ☐ (3) Somewhat bothersome
   - ☐ (2) Very bothersome
   - ☐ (1) A constant bother
4. How would you rate the function of your bladder?
   - ☐ (6) Excellent
   - ☐ (5) Very good
   - ☐ (4) Good
   - ☐ (3) Fair
   - ☐ (2) Poor
   - ☐ (1) Terrible
5. Compared to others your age, is your bladder function…
   - ☐ (5) Much better
   - ☐ (4) Somewhat better
   - ☐ (3) About the same
   - ☐ (2) Somewhat worse
   - ☐ (1) Much worse
6. When you feel the need to pee, once you get to the bathroom how well does "getting done what you need to do" happen for you?
   - ☐ (4) I am just in and out and on with my day
   - ☐ (3) I take care of things pretty well
   - ☐ (2) It can be more of a chore than I would like
   - ☐ (1) I dread when I need to pee

7. When it comes to my bladder…

| ☐1 | ☐2 | ☐3 | ☐4 | ☐5 | ☐6 | ☐7 | ☐8 | ☐9 | ☐10 |
|---|---|---|---|---|---|---|---|---|---|
| It controls me | | | | It is a give and take relationship | | | | | I control it |

8. How easy or difficult are each of the following

| | Very easy (6) | Easy (5) | Somewhat easy (4) | Somewhat difficult (3) | Difficult (2) | Very difficult (1) |
|---|---|---|---|---|---|---|
| a. When you feel the need to pee, how easy or difficult is it to hold it? | ☐ | ☐ | ☐ | ☐ | ☐ | ☐ |
| b. When you feel the need to pee, how easy or difficult is it to start peeing? | ☐ | ☐ | ☐ | ☐ | ☐ | ☐ |
| c. When you feel the need to pee, how easy or difficult is it to completely empty your bladder? | ☐ | ☐ | ☐ | ☐ | ☐ | ☐ |

9. How much does fear of embarrassment restrict your activities?
   - ☐ (4) Never
   - ☐ (3) Rarely
   - ☐ (2) Sometimes
   - ☐ (1) Usually
10. How often do you worry about your bladder, such as worrying about accidental leakage, being able to make it to the bathroom in time, etc.?
    - ☐ (5) Never
    - ☐ (4) Rarely
    - ☐ (3) Sometimes
    - ☐ (2) Usually
    - ☐ (1) All the time

*Adaptive behaviors.*

11. How often do you use a liner, pad, or absorbent underwear, in case of accidental urine leakage?
    - ☐ (1) None of the time → **SKIP TO QUESTION 12**
    - ☐ (2) A little of the time
    - ☐ (3) Some of the time
    - ☐ (4) Most of the time
    - ☐ (5) All the time

11a How much confidence does this give you?
- ☐ (1) Not much at all
- ☐ (2) A little
- ☐ (3) Some
- ☐ (4) A lot
- ☐ (5) Complete confidence

→ **AFTER ANSWERING QUESTION 12a SKIP TO QUESTION 13**

12. How often do you carry supplies such as panty liners, pads, or extra underwear etc. with you because of your bladder?
    - ☐ (1) Never → SKIP TO QUESTION 13
    - ☐ (2) Rarely
    - ☐ (3) Sometimes
    - ☐ (4) Usually
    - ☐ (5) Won't leave home without it

12a How much does having these things available give you the confidence to do the things you need or want to do?
- ☐ (1) Not much at all
- ☐ (2) A little
- ☐ (3) Some
- ☐ (4) A lot
- ☐ (5) Complete confidence

13. How often is finding out where the bathrooms are one of the first things you do when you go someplace?
    - ☐ (1) None of the time → **SKIP TO QUESTION 14**
    - ☐ (2) A little of the time
    - ☐ (3) Some of the time
    - ☐ (4) Most of the time
    - ☐ (5) All the time

13a How much confidence does this give you?
- ☐ (1) Not much at all
- ☐ (2) A little
- ☐ (3) Some
- ☐ (4) A lot
- ☐ (5) Complete confidence

→ **AFTER ANSWERING SKIP TO QUESTION 15**

14. How often do you stay as close to a bathroom as possible when you are away from home?
    - ☐ (1) None of the time → **SKIP TO QUESTION 15**
    - ☐ (2) A little of the time
    - ☐ (3) Some of the time
    - ☐ (4) Most of the time
    - ☐ (5) All the time

14a How much confidence does this give you?
- ☐ (1) Not much at all
- ☐ (2) A little
- ☐ (3) Some
- ☐ (4) A lot
- ☐ (5) Complete confidence

Bladder Function Indices

The next set of questions is about things you may have experienced. Before starting the questions, please look at each of the following descriptions of bladder-related experiences.

- Urinary tract infections or bladder infections that you had to take antibiotics for
- Had times when you peed more often than usual or expected
- A sudden and urgent need to pee, that "gotta go" feeling that you just had to go
- Accidental leakage of urine
- Discomfort, pain, pressure, or burning in your bladder when peeing
- Trouble starting to pee, or completely emptying your bladder, or dribbling a few drops after you finish peeing

15. Have you ever been told by a healthcare provider that you had a urinary tract infection (UTI)?
    - ☐ No, I have never had a UTI in my life → **SKIP TO QUESTION 16**
    - ☐ Yes, I have had at least one in my life but not in the past year
    - ☐ Yes, I have had a UTI in the past year

15a Have you ever in your life had 3 or more UTIs in a year?
- ☐ No → SKIP TO QUESTION 16
- ☐ Yes, but not in the past year
- ☐ Yes, and it was in the past year

15b Which of the following best describes your UTIs? (Choose only one)
- ☐ (1) Constant: more or less the same for the entire year
- ☐ (2) Intermittent: sometimes it is better and other times it is worse
- ☐ (3) Sporadic: it happens every once in a while

15c When you had UTIs, does your bladder get back to your normal or baseline?
- ☐ (7) Very quickly
- ☐ (6) Quickly
- ☐ (5) Somewhat quickly
- ☐ (4) Somewhat slowly
- ☐ (3) Slowly
- ☐ (2) Very slowly
- ☐ (1) It never seems to get completely better

15d Overall, how much has this interfered with your life?
- ☐ (5) Not at all
- ☐ (4) A little bit
- ☐ (3) Some
- ☐ (2) A lot
- ☐ (1) Completely

16. Have you ever had times when you peed more often than usual, either during day/waking hours or night/sleeping hours? Please do NOT count or consider times when this was a result of having a UTI.
- ☐ No, not even once → **SKIP TO QUESTION 17**
- ☐ Yes, but not in the past year
- ☐ Yes, this has happened in the past year

16a Which of the following best describes your experiences with peeing more often than usual? (Choose only one)
- ☐ (1) Constant: more or less the same for the entire year
- ☐ (2) Intermittent: sometimes it is better and other times it is worse
- ☐ (3) Sporadic: it happens every once in a while

16b When this happens, would you say that your bladder gets back to your normal or baseline?
- ☐ (7) Very quickly
- ☐ (6) Quickly
- ☐ (5) Somewhat quickly
- ☐ (4) Somewhat slowly
- ☐ (3) Slowly
- ☐ (2) Very slowly
- ☐ (1) It never seems to get completely better

16c Overall, how much has this need to pee more often than usual interfered with your life?
- ☐ (5) Not at all
- ☐ (4) A little bit
- ☐ (3) Some
- ☐ (2) A lot
- ☐ (1) Completely

16d Compared to 1 year ago, is your experience now with peeing more often than usual?
- ☐ (5) Much better now than 1 year ago
- ☐ (4) Somewhat better now than 1 year ago
- ☐ (3) About the same as 1 year ago
- ☐ (2) Somewhat worse now than 1 year ago
- ☐ (1) Much worse now than 1 year ago

17. Have you ever experienced a sudden and urgent need to pee, that "gotta go" feeling that you just had to go? Please do NOT count or consider times when this was a result of having a UTI.
- ☐ No, not even once → **SKIP TO QUESTION 18**
- ☐ Yes, but not in the past year
- ☐ Yes, this has happened in the past year

17a Which of the following best describes your experiences with the sudden and urgent need to pee? (Choose only one)
- ☐ (1) Constant: more or less the same for the entire year
- ☐ (2) Intermittent: sometimes it is better and other times it is worse
- ☐ (3) Sporadic: it happens every once in a while

17b When this occurs, would you say that your bladder gets back to your normal or baseline?
- ☐ (7) Very quickly
- ☐ (6) Quickly
- ☐ (5) Somewhat quickly
- ☐ (4) Somewhat slowly
- ☐ (3) Slowly
- ☐ (2) Very slowly
- ☐ (1) It never seems to get completely better

17c Overall, how much has this interfered with your life?
- ☐ (5) Not at all
- ☐ (4) A little bit
- ☐ (3) Some
- ☐ (2) A lot
- ☐ (1) Completely

17d Compared to 1 year ago, is your experience with the sudden and urgent need to pee better or worse?
- ☐ (5) Much better now than 1 year ago
- ☐ (4) Somewhat better now than 1 year ago
- ☐ (3) About the same as 1 year ago
- ☐ (2) Somewhat worse now than 1 year ago
- ☐ (1) Much worse now than 1 year ago

18. Have you ever accidentally leaked urine, even just a drop or 2? Please do NOT count or consider times when this was a result of having a UTI.

☐ No, not even once → **SKIP TO QUESTION 19**

☐ Yes, but not in the past year

☐ Yes, this has happened in the past year

18a Which of the following best describes your experiences with accidentally leaking urine? (Choose only one)

☐ (1) Constant: more or less the same for the entire year

☐ (2) Intermittent: sometimes it is better and other times it is worse

☐ (3) Sporadic: it happens every once in a while

18b When this occurs, would you say that your bladder gets back to your normal or baseline?

☐ (7) Very quickly

☐ (6) Quickly

☐ (5) Somewhat quickly

☐ (4) Somewhat slowly

☐ (3) Slowly

☐ (2) Very slowly

☐ (1) It never seems to get completely better

18c Overall, how much has this accidental urine leakage interfered with your life?

☐ (5) Not at all

☐ (4) A little bit

☐ (3) Some

☐ (2) A lot

☐ (1) Completely

18d Compared to 1 year ago, is your experience with accidentally leaking urine?

☐ (5) Much better now than 1 year ago

☐ (4) Somewhat better now than 1 year ago

☐ (3) About the same as 1 year ago

☐ (2) Somewhat worse now than 1 year ago

☐ (1) Much worse now than 1 year ago

19. Have you ever experienced any of these sensations in your pelvis or lower abdomen related to peeing or holding urine? Please do NOT count or consider times when this was a result of having a UTI.

- A cramping, aching, or stabbing sensation
- Discomfort or pressure
- Burning

☐ No, not even once → **SKIP TO QUESTION 20**

☐ Yes, but not in the past year

☐ Yes, this has happened in the past year

19a Which of the following best describes your experiences with these sensations in your pelvis or lower abdomen related to peeing or holding urine? (Choose only one)

☐ (1) Constant: more or less the same for the entire year

☐ (2) Intermittent: sometimes it is better and other times it is worse

☐ (3) Sporadic: it happens every once in a while

19b When this occurs, would you say that your bladder gets back to your normal or baseline?

☐ (7) Very quickly

☐ (6) Quickly

☐ (5) Somewhat quickly

☐ (4) Somewhat slowly

☐ (3) Slowly

☐ (2) Very slowly

☐ (1) It never seems to get completely better

19c Overall, how much has this interfered with your life?

☐ (5) Not at all

☐ (4) A little bit

☐ (3) Some

☐ (2) A lot

☐ (1) Completely

19d Compared to 1 year ago, is this better or worse?

☐ (5) Much better now than 1 year ago

☐ (4) Somewhat better now than 1 year ago

☐ (3) About the same as 1 year ago

☐ (2) Somewhat worse now than 1 year ago

☐ (1) Much worse now than 1 year ago

20. The following is a list of things that can happen when a person pees, have you ever experienced any of them? Please do NOT count or consider times when this was a result of having a UTI.

- Trouble or difficulty starting to pee
- Feel like you are not completely emptying your bladder when you have finished peeing (feel like you still need to pee some more, but nothing comes out)
- When you pee it flows slowly (just seems to trickle out) or sprays
- Your urine will start and stop while you are trying to pee
- Dribbling at least a few drops after you think you have finished peeing

☐ No, not even once → **SKIP TO END**

☐ Yes, but not in the past year

☐ Yes, this has happened in the past year

20a Which of the following best describes your experiences with peeing? (Choose only one)

☐ (1) Constant: more or less the same for the entire year

☐ (2) Intermittent: sometimes it is better and other times it is worse

☐ (3) Sporadic: it happens every once in a while

20b When this occurs, would you say that your bladder gets back to your normal or baseline?

☐ (7) Very quickly

☐ (6) Quickly

☐ (5) Somewhat quickly

☐ (4) Somewhat slowly

☐ (3) Slowly

☐ (2) Very slowly

☐ (1) It never seems to get completely better

20c Overall, how much has this interfered with your life?

☐ (5) Not at all

☐ (4) A little bit

☐ (3) Some

☐ (2) A lot

☐ (1) Completely

20d Compared to 1 year ago, is your peeing better or worse?

☐ (5) Much better now than 1 year ago

☐ (4) Somewhat better now than 1 year ago

☐ (3) About the same as 1 year ago

☐ (2) Somewhat worse now than 1 year ago

☐ (1) Much worse now than 1 year ago

## Supplemental material 2 — Scoring methodology for BHS-3 with ABA & BFI

The following material specifies the content and scoring for each of the following:

- Abbreviated Bladder Health Scale (BHS-3)
- Adaptive Behavior Adjustment (ABA)
- Bladder Function Indices (BFIs)

The BHS and BFI are both scored using a transformed sum approach to yield scale and index scores that range from 0 to 100 with higher values indicating better health/functioning. Use of the transformed sum alleviates the need for imputation relative to item nonresponse. The ABA does not use transformed scoring but modifies the transformed sum of the BHS.

The items included for scoring each A-BHS are included in Supplemental Table 1 below.

**SUPPLEMENTAL TABLE 1**
**Scoring the BHS-3**

| Scale | Items included (see Appendix A for how responses are coded) | Minimum number of items that must be answered[a] |
|---|---|---|
| Global | 1, 2, 3, 4, 5, 7 | 4 |
| Holding | 8a, 9, 10 | 2 |
| Perceived efficacy | 6, 8b, 8c | 2 |

*BHS*, Bladder Health Scales.

To calculate a transformed score for the BHS, the following steps must be followed. Supplemental Table 2 provides illustrative examples of calculating transformed scores for the BHS Global Scale. Item response values are shown in Appendix A within parentheses.

1. Calculate the sum of the answered items.

$$Simple\ sum = \sum Answered\ item\ response\ values$$

2. Determine the minimum and maximum values based on the items answered by the respondent.
   a. $Adjusted\ min = \sum Minimum\ possible\ item\ response\ values\ of\ answered\ items$
   b. $Adjusted\ max = \sum Maximum\ possible\ item\ response\ values\ of\ answered\ items$
   c. $Range = Adjusted\ max - Adjusted\ min$
3. Calculate the transformed sum score.

$$Transformed\ Sum = \left(\frac{Simple\ sum - Adjusted\ min}{Range}\right) * 100$$

**SUPPLEMENTAL TABLE 2**
**Example of transformed scoring for the Global Scale**

| Item | 1 | 2 | 3 | 4 | 5 | 7 | Simple sum | Adjusted minimum/ maximum/range | Transformed sum |
|---|---|---|---|---|---|---|---|---|---|
| Item minimum—maximum values | 1—6 | 1—6 | 1—5 | 1—6 | 1—5 | 1—10 | | | |
| Respondent 1 | 3 | 2 | 2 | 4 | 4 | 8 | 23 | Minimum: 6<br>Maximum: 38<br>Range: 32 | 53 |
| Respondent 2 | 2 | | 4 | 5 | | 3 | 14 | Minimum: 4<br>Maximum: 27<br>Range: 23 | 43 |
| Respondent 3 | 3 | 4 | 2 | 2 | 4 | | 15 | Minimum: 5<br>Maximum: 28<br>Range: 23 | 43 |
| Respondent 4 | 4 | 6 | 4 | 5 | | 9 | 28 | Minimum: 5<br>Maximum: 33<br>Range: 28 | 82 |
| Respondent 5 | 5 | | 4 | | 4 | 7 | 20 | Minimum: 4<br>Maximum: 26<br>Range: 22 | 73 |
| Respondent 6 | 2 | 1 | 4 | | | | Not scored (only 3 items answered) | | |

**ABA scoring**

The ABA reflects 2 types of adaptive behavior, AB1 and AB2, both of which include conditional scoring steps. The items included for scoring of each type of adaptive behavior are included in Supplemental Table 5 below with item and conditionally specific scoring instruction.

**SUPPLEMENTAL TABLE 5**
**Adaptive behavior scoring**

| | Items | Conditional step 1 | Conditional step 2 | Scoring |
|---|---|---|---|---|
| AB1 | 11, 11a, 12, 12a | If 11≥2 | N/A | AB1 score=11+11a |
| | | If 11=1 (none) →conditional step 2 | If 12≥2 | AB1 score=12+12a |
| | | If 11=1 (none) →conditional step 2 | If 12=1 (none) | AB1 score=2 |
| AB2 | 13, 13a, 14, 14a | If 13≥2 | N/A | AB2 score=13+13a |
| | | If 13=1 (none) →conditional step 2 | If 14≥2 | AB2 score=14+14a |
| | | If 13=1 (none) →conditional step 2 | If 14=1 (none) | AB2 score=2 |
| | Total adaptive score | | Score=11-(AB1+AB2)/2 | |

The Adaptive Behavior Adjustment (ABA) is to be applied only to the BHS scores. Application of the ABA occurs by changing the Transformed Sum scoring process slightly to accommodate the adjustment. Supplemental Table 6 demonstrates the application of the adjusted scoring process. It is critical to note that a person who has a 0 for the transformed sum score will NOT necessarily have a 0 for the adaptive behavior adjusted transformed summation.

*AB*, adaptive behavior; *N/A*, not applicable.

$$Transformed\ Sum = \left(\frac{(Simple\ sum * Adaptive\ Score) - (Adjusted\ min)}{(9 * Adjusted\ max) - (Adjusted\ min)}\right) * 100$$

**SUPPLEMENTAL TABLE 6**
**Application of the Adaptive Behavior Adjustment to Global Scale example found in Supplemental Table 2**

| Respondent | Simple sum | Adaptive score | Adjusted minimum/ maximum | Adaptive behavior adjusted transformed sum | Unadjusted transformed sum (difference in score based on adjustment) |
|---|---|---|---|---|---|
| 1 | 23 | 2 | Minimum: 6<br>Maximum: 38 | 12=(((23*2)-6)/((9*38)-6))*100 | 53 (−41) |
| 2 | 14 | 8 | Minimum: 4<br>Maximum: 27 | 45=(((14*8)-4)/((9*27)-4))*100 | 43 (2) |
| 3 | 15 | 3 | Minimum: 5<br>Maximum: 28 | 16=(((15*3)-5)/((9*28)-5))*100 | 43 (−27) |
| 4 | 28 | 9 | Minimum: 5<br>Maximum: 33 | 85=(((28*9)-5)/((9*33)-5))*100 | 82 (3) |
| 5 | 20 | 1 | Minimum: 4<br>Maximum: 26 | 7=(((20*1)-4)/((9*26)-4))*100 | 73 (−66) |

**BFI scoring**

The items included for scoring of each BFI are included in Supplemental Table 3 below with specific scoring instructions when certain conditions are met.

**SUPPLEMENTAL TABLE 3**

**BFI items**

| Index | Conditional instructions for scoring | Simple sum items | Minimum number of simple sum items that must have a value[a] |
|---|---|---|---|
| UTI | If 15 or 15a are "No" then set 15b=4, 15c=8, 15d=6 | 15b, 15c, 15d | 2 |
| Frequency | If 16 is "No" then set 16a=4, 16b=8, 16c=6, 16d=6 | 16a, 16b, 16c, 16d | 3 |
| Sensation | If 17 is "No" then set 17a=4, 17b=8, 17c=6, 17d=6 | 17a, 17b, 17c, 17d | 3 |
| Continence | If 18 is "No" then set 18a=4, 18b=8, 18c=6, 18d=6 | 18a, 18b, 18c, 18d | 3 |
| Comfort | If 19 is "No" then set 19a=4, 19b=8, 19c=6, 19d=6 | 19a, 19b, 19c, 19d | 3 |
| Emptying | If 20 is "No" then set 20a=4, 20b=8, 20c=6, 20d=6 | 20a, 20b, 20c, 20d | 3 |

The process for calculating BFI transformed scores follows the same process as calculation of transformed scores for the BHS. Illustrative examples of transformed score calculation for the BFI Frequency Index are provided in Supplemental Table 4.

*BFI*, Bladder Function Index; *UTI*, urinary tract infection.

[a] If the minimum number of items is not met, the scale should not be scored.

1. Calculate the sum of the answered items.

$$Simple\ sum = \sum Answered\ item\ response\ values$$

2. Determine the minimum and maximum values based on the items answered by the respondent. Note that due to the conditional instructions for scoring (Supplemental Table 3), the maximum value for an item will be one more than the maximum response value shown in Appendix A.
   a. $Adjusted\ min = \sum Minimum\ possible\ item\ response\ values\ of\ answered\ items$
   b. $Adjusted\ max = \sum Maximum\ possible\ item\ response\ values\ of\ answered\ items$
   c. $Range = Adjusted\ max - Adjusted\ min$
3. Calculate the transformed sum score.

$$Transformed\ Sum = \left(\frac{Simple\ sum - Adjusted\ min}{Range}\right) * 100$$

The process for calculating BFI transformed scores follows the same process as calculation of transformed scores for the BHS.

Supplemental Table 4 provides examples of transformed sum scores for the Bladder Function Frequency Index. The transformed sum is calculated using the questions identified in the Simple Sums Items column.

**SUPPLEMENTAL TABLE 4**

**Example of transformed sum scoring for the frequency index**

| | Conditional item | Simple sum items | | | | | | |
|---|---|---|---|---|---|---|---|---|
| Item | 16[a] | 16a | 16b | 16c | 16d | Simple sum | Adjusted minimum/ maximum/range | Transformed sum |
| Item minimum—maximum values | | 1—4 | 1—8 | 1—6 | 1—6 | | | |
| Respondent 1 | 1 | 4[b] | 8[b] | 6[b] | 6[b] | 24 | Minimum: 4<br>Maximum: 24<br>Range: 20 | 100 |
| Respondent 2 | 2 | 2 | 4 | 5 | 3 | 14 | Minimum: 4<br>Maximum: 24<br>Range: 20 | 50 |
| Respondent 3 | 2 | 1 | | 2 | 4 | 7 | Minimum: 3<br>Maximum: 16<br>Range: 13 | 31 |
| Respondent 4 | 2 | 3 | 4 | 3 | | 10 | Minimum: 3<br>Maximum: 18<br>Range: 15 | 46 |
| Respondent 5 | 2 | 2 | 4 | | | Not scored (only 2 items answered) | | |

[a] Item 16 is not included in the simple summation.[b] Default maximum values based on response to item 16 and conditional instructions for scoring.
